# The halophilic alkalithermophile *Natranaerobius thermophilus* adapts to multiple environmental extremes using a large repertoire of Na^+^(K^+^)/H^+^ antiporters

**DOI:** 10.1111/j.1365-2958.2009.06845.x

**Published:** 2009-08-28

**Authors:** Noha M Mesbah, Gregory M Cook, Juergen Wiegel

**Affiliations:** 1 Department of Microbiology, University of GeorgiaAthens, GA 30602, USA.; 2 Department of Microbiology and Immunology, University of OtagoDunedin, New Zealand

## Abstract

*Natranaerobius thermophilus* is an unusual extremophile because it is halophilic, alkaliphilic and thermophilic, growing optimally at 3.5 M Na^+^, pH^55°C^ 9.5 and 53°C. Mechanisms enabling this tripartite lifestyle are essential for understanding how microorganisms grow under inhospitable conditions, but remain unknown, particularly in extremophiles growing under multiple extremes. We report on the response of *N. thermophilus* to external pH at high salt and elevated temperature and identify mechanisms responsible for this adaptation. *N. thermophilus* exhibited cytoplasm acidification, maintaining an unanticipated transmembrane pH gradient of 1 unit over the entire extracellular pH range for growth. *N. thermophilus* uses two distinct mechanisms for cytoplasm acidification. At extracellular pH values at and below the optimum, *N. thermophilus* utilizes at least eight electrogenic Na^+^(K^+^)/H^+^ antiporters for cytoplasm acidification. Characterization of these antiporters in antiporter-deficient *Escherichia coli* KNabc showed overlapping pH profiles (pH 7.8–10.0) and Na^+^ concentrations for activity (*K*_0.5_ values 1.0–4.4 mM), properties that correlate with intracellular conditions of *N. thermophilus*. As the extracellular pH increases beyond the optimum, electrogenic antiport activity ceases, and cytoplasm acidification is achieved by energy-independent physiochemical effects (cytoplasmic buffering) potentially mediated by an acidic proteome. The combination of these strategies allows *N. thermophilus* to grow over a range of extracellular pH and Na^+^ concentrations and protect biomolecules under multiple extreme conditions.

## Introduction

Extremophilic microorganisms are excellent models for the study and characterization of novel physiologies and biochemical pathways, and are also models for possible extraterrestrial life. To date, the majority of studies have focused on extremophiles growing at one environmental extreme, such as high temperature, high salinity or acid/alkaline pH. Microorganisms that cope with two extremes (e.g. alkalithermophiles, acidothermophiles) are well documented (Segerer *et al*., 1988; Schleper *et al*., 1995; Cook *et al*., 1996; Prowe *et al*., 1996; Peddie *et al*., 1999; Olsson *et al*., 2003; Huber and Prangishvili, 2006), but to our knowledge, bacteria growing under three environmental extremes have not been studied in detail. Recent work from our laboratory has described the isolation and characterization of novel anaerobic bacteria growing at a unique combination of extreme conditions (Mesbah *et al*., 2007; Mesbah and Wiegel, 2009). These new bacterial isolates are obligately anaerobic, chemoorganotrophic and grow optimally with a doubling time of a few hours under the combined conditions of elevated temperature (≥ 50°C), alkaline pH (pH^55°C^ ≥ 8.5, equivalent to pH^25°C^ 9.5), and elevated salt concentration (≥ 3.2 M Na^+^) [The superscript denotes the temperature at which the pH was measured and the pH meter calibrated. SeeWiegel (1998) for more details]. These microorganisms are thermophilic, alkaliphilic and halophilic, and these growth conditions place them into the unusual group of ‘poly extremophiles’, termed the halophilic alkalithermophiles. The taxa of halophilic alkalithermophiles isolated thus far represent a novel order within the *Firmicutes,* the *Natranaerobiales* (Mesbah *et al*., 2007; Mesbah and Wiegel, 2009). Their high pH and temperature optima/maxima for growth distinguish them from other anaerobic halophiles within the *Firmicutes*.

One of these halophilic alkalithermophiles, *Natranaerobius thermophilus*, was isolated from sediments of the alkaline, hypersaline lakes of the Wadi An Natrun, Egypt (Mesbah *et al*., 2007). *N. thermophilus* grows between 35°C and 56°C, with an optimum at 53°C. The pH^55°C^ range for growth is 8.3–10.6, with an optimum at pH^55°C^ 9.5 and no growth at pH^55°C^ 8.2 or below or pH^55°C^ 10.8 or above. This corresponds to pH^25°C^ 9.3–11.2, with an optimum at pH^25°C^ 10.5. At optimum pH^55°C^ and temperature, *N. thermophilus* grows in the Na^+^ concentration range of 3.1–4.9 M and optimally between 3.3 and 3.9 M Na^+^. It is a non-motile and strictly anaerobic chemoorganotroph. As *N. thermophilus* is a halophile, alkaliphile and thermophile, its physiological and bioenergetic characteristics must be compatible with these complex and extreme growth conditions. As an alkaliphile, *N. thermophilus* must be able to acidify the cytoplasm in order to preserve the functional and structural integrity of cytoplasmic proteins supporting growth. As a thermophilic and halophilic bacterium, *N. thermophilus* is confronted with the problem of passive permeation of H^+^ and Na^+^ through the cytoplasmic membrane, processes that increase with high temperature and elevated Na^+^ concentration (Vossenberg *et al*., 1999; Konings *et al*., 2002). Na^+^ toxicity occurs due to an increase in intracellular Na^+^ concentrations. While this increase can range from 20 mM in non-halophiles to over 3 M in extremely halophilic archaea, all prokaryotes maintain intracellular Na^+^ concentrations lower than the extracellular concentration (Padan and Krulwich, 2000). Increased proton permeability of the cytoplasmic membrane compromises both cytoplasmic acidification mechanisms that are critical for survival at alkaline pH, and the ability to maintain a membrane potential.

Given these considerations, the bioenergetics of *N. thermophilus* becomes an intriguing problem. Previous studies have investigated response to alkaline pH in alkaliphiles (Sturr *et al*., 1994; Aono *et al*., 1997; Kitada *et al*., 2000), and alkalithermophiles (Cook *et al*., 1996; Olsson *et al*., 2003). These microorganisms exhibited intracellular pH regulation over their extracellular pH ranges for growth, and the transmembrane pH gradient (ΔpH, pH_out_-pH_in_) decreased and, in the case of the anaerobic alkalithermophile *Clostridium paradoxum*, collapsed under extreme alkaline stress as well as the minimal pH value where growth ceased. The limiting factor for growth at extreme alkaline pH appears to be the reduced capacity for cytoplasm acidification. However, the precise mechanisms allowing intracellular pH homeostasis have not been investigated in detail.

We report on the biochemical and molecular strategies used by *N. thermophilus* to combat extreme pH at high salinity and temperature. *N. thermophilus* exhibited cytoplasm acidification, but has the unusual characteristic of ΔpH homeostasis that is different from what has been previously reported for other alkaliphiles and alkalithermophiles (Sturr *et al*., 1994; Cook *et al*., 1996; Aono *et al*., 1997; Olsson *et al*., 2003). Intracellular pH in *N. thermophilus* was maintained at a constant 1 unit below the extracellular pH and the ΔpH did not collapse, even under extreme alkaline stress. In order to determine the mechanism of this unique cytoplasm pH regulation, the genome sequence of *N. thermophilus* was analysed for putative cation/proton antiporter genes. Of the 12 putative cation/proton antiporters identified, 8 were functionally expressed in the triple antiporter-deficient *Escherichia coli* KNabc. Various aspects of the biological activity and transcription of these antiporters were characterized, and their roles in physiology of growth at high Na^+^ concentration and pH are discussed.

## Results

### *N. thermophilus* exhibits active and passive mechanisms of cytoplasm pH acidification

**Fig. 1:**
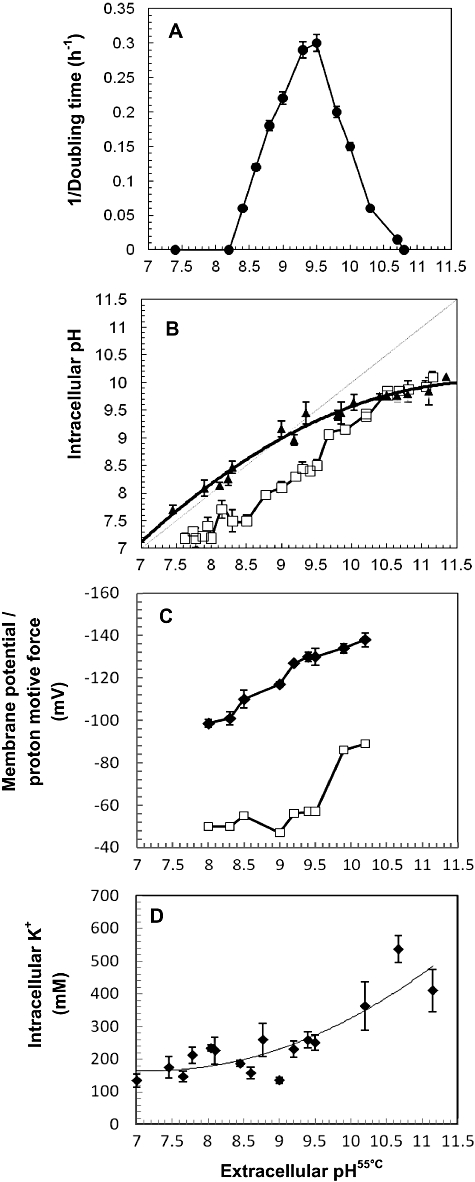
Bioenergetic parameters in *N. thermophilus*. A. Effect of external pH^55°C^ on growth of *N. thermophilus* in batch culture. B. Effect of external pH on intracellular pH in sucrose-energized cell suspensions (□) and non-energized cell suspensions (▴). The diagonal line represents absence of a ΔpH. C. Effect of external pH^55°C^ on Δψ (♦) and proton motive force (□) in energized cell suspensions of *N. thermophilus*. D. Effect of external pH^55°C^ on intracellular K^+^ concentration in energized cell suspensions of *N. thermophilus*. All values reported are the mean of three independent experiments; the standard error associated with the determinations is shown.

The obligately anaerobic *N. thermophilus* is able to grow in the pH^55°C^ range 8.3–10.6 and no growth is observed below pH^55°C^ 8.2 and above pH^55°C^ 10.8 (Fig. 1A). The main organic fermentation products from sucrose at pH^55°C^ 9.5, 53°C and 3.5 M Na^+^ are acetate and formate (Mesbah *et al*., 2007). The effect of external pH on intracellular pH was examined in sucrose-energized cell suspensions of *N. thermophilus* over the pH^55°C^ range 8.0–11.0 (Fig. 1B). At the optimal pH^55°C^ for growth of 9.5, 3.3 M Na^+^ and 53°C, *N. thermophilus* maintained a ΔpH of approximately 1 unit (acid_in_). This ΔpH of 1 unit was due to active cytoplasm acidification as the gradient (measured at optimal extracellular conditions) was collapsed with inhibitors that dissipate the ΔpH [e.g. 3,3′,4′,5-tetrachlorosalicylanilide, nigericin (Fig. S1A)], and the ΔpH was absent in non-energized suspensions of *N. thermophilus* (see below). As the external pH^55°C^ was increased from 8.0 to 11.0, the intracellular pH^55°C^ increased from 7.2 to 9.9 (Fig. 1B). Strikingly, the ΔpH across the cell membrane did not collapse, even at the upper and lower pH^55°C^ boundaries for growth. This suggests that cessation of growth at more alkaline pH values is not due to a decrease in ΔpH as has been reported for other alkaliphiles and alkalithermophiles (Sturr *et al*., 1994; Cook *et al*., 1996; Olsson *et al*., 2003). To investigate the nature of this ΔpH homeostasis at high extracellular pH^55°C^ values, the experiment was repeated with non-energized cells. The ΔpH of non-energized cells was close to zero until the external pH^55°C^ was > 9.5, at which point the same pattern of cytoplasm acidification of energized cells was observed. This ΔpH was only partially collapsed by protonophores and ionophores (Fig. S1B), suggesting an energy-independent component to ΔpH homeostasis (e.g. cytoplasmic buffering capacity).

The electrochemical membrane potential (Δψ) of energized cell suspensions varied between −99 and −138 mV (Fig. 1C) and the overall proton motive force (*pmf*) was between −50 and −89 mV (Fig. 1C) as a result of the oppositely orientated *Z*ΔpH. Despite the low overall *pmf*, the phosphorylation potential was maintained between −477 and −491 mV (data not shown). Intracellular K^+^ concentrations in energized cell suspensions remained constant at approximately 250 mM below extracellular pH^55°C^ 9.5, but increased sharply at more alkaline pH values reaching 540 mM at pH^55°C^ 10.6 (Fig. 1D). The intracellular Na^+^ concentration in both exponentially growing and energized cells was 8 mM at extracellular pH^55°C^ 9.5, and increased to 33 mM at pH^55°C^ 10.5 (data not shown).

### N. thermophilus harbours a large repertoire of Na^+^(K^+^)/H^+^ antiporters in its genome

To determine the mechanism for cytoplasm acidification in *N. thermophilus*, the genome sequence was analysed for transport systems that could participate in cytoplasm acidification [e.g. Na^+^(K^+^)/H^+^ antiporters] using a semi-automated pipeline (Ren *et al*., 2007). Twelve genes with significant homology to Na^+^/H^+^ exchangers were identified by these analyses (Table S1). Eight of these genes had homology to members of the NhaC Na^+^/H^+^ antiporter family (Saier *et al*., 2006), and were encoded alone and did not appear to be part of an operon. Three genes were 38–48% identical to Na^+^/H^+^ exchanger proteins from ‘*Alkaliphilus metalliredigens’* and ‘*Alkaliphilus oremlandii’*, two obligately anaerobic, non-halophilic and moderately alkaliphilic bacteria (Ye *et al*., 2004; Fisher *et al*., 2008). Gene *nt-Nha* had 35% identity to the *shaA* (*mrPA*) gene of *Clostridium tetani*. The Mrp proteins belong to the monovalent cation/proton antiporter-3 protein family. This family is composed of multi-component Na^+^/H^+^ and K^+^/H^+^ antiporters encoded by operons of six or seven genes, and all genes are required for full function in Na^+^ and alkali resistance (Ito *et al*., 2000). Sequence analysis of the regions surrounding gene *nt-Nha*, however, did not show that it was part of an operon. This indicates that gene *nt-Nha* does not encode a subunit of an Mrp system, but rather a mono-subunit antiporter.

### *N. thermophilus* antiporter genes complement the Na^+^ and alkali sensitivities of *E. coli*KNabc

**Fig. 2:**
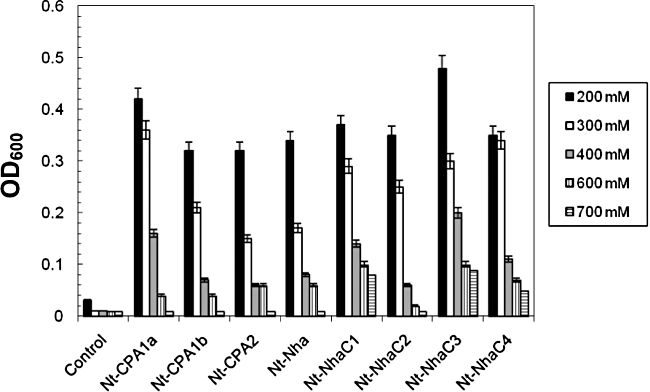
Effect of NaCl concentration on the growth of *E. coli* KNabc transformants of *N. thermophilus* antiporters. Transformants with vector control pGEM-3Zf(+), or expressing *N. thermophilus* antiporter genes, were grown anaerobically on LBK medium, pH^37°C^ 7.5, containing indicated concentrations of NaCl.

To verify the function of the predicted antiporter genes, the individual genes were expressed heterolougsly in antiporter deficient strain *E. coli* KNabc (Δ*nhaA*Δ*nhaB*Δ*chaA*). *E. coli* strain KNabc is sensitive to NaCl concentrations of 200 mM and above and pH^37°C^ values > 8.0 (Goldberg *et al*., 1987). Of the 12 cloned putative antiporter genes identified, 8 were able to complement the Na^+^-sensitive phenotype of strain KNabc (Fig. 2). The KNabc-Nt-NhaC1, -Nt-NhaC3 and -Nt-NhaC4 transformants exhibited Na^+^ resistance up to 700 mM at pH^37°C^ 7.5, while the remaining transformants supported resistance at up to 600 mM NaCl (Fig. 2). None of the transformants supported growth at NaCl concentrations > 750 mM. The same eight *N. thermophilus* antiporter genes supported modest alkali-resistance allowing growth at pH^37°C^ values up to 8.5 in the absence of added NaCl. The remaining four genes did not complement the Na^+^-sensitivity phenotype of *E. coli* KNabc, but were able to complement the K^+^-uptake deficiency of *E. coli* TK2420 (Kdp^-^, Kup^-^, Trk^-^), indicating that they have potential roles in transport of K^+^ into the cell (Fig. S2). Genomic analyses showed that these four genes are encoded alone and do not appear to be part of an operon or any other system.

### *N. thermophilus* antiporters catalyse Na^+^/H^+^ and K^+^/H^+^ antiport at high pH with a wide range of *K*_0.5_ for cations

Biochemical assays with inverted membrane vesicles of strain KNabc expressing the individual *N. thermophilus* antiporters were carried out to determine antiport activity with the fluorescent dye acridine orange (Goldberg *et al*., 1987) (see *Experimental procedures* for details). Initial experiments were conducted at pH^37°C^ 8.5 in order to simulate the intracellular pH measured in energized cell suspensions of *N. thermophilus* (Fig. 1B). When inverted membrane vesicles of strain KNabc were incubated with Trizma-succinate, strong quenching of acridine orange was observed indicating the formation of a ΔpH. The signal was reversed by the addition of NaCl indicating Na^+^/H^+^ antiporter activity. With the exception of Nt-NhaC3, all *N. thermophilus* antiporter proteins exhibited significant Na^+^-dependent antiport activity, with a dequenching percentage as high as 77% for Nt-CPA1a (Table 1). K^+^/H^+^ antiport activity was observed for all eight putative antiporters in the concentration range between 3 and 31 mM K^+^. Nt-NhaC3 exhibited solely K^+^/H^+^ antiport activity and did not show any Na^+^-dependent activities using a range of Na^+^ concentrations (2–100 mM) (Table 1).

**Table 1:** Monovalent cation/proton antiport activity in inverted membrane vesicles of antiporter-expressing *E. coli* KNabc transformants.

	% Dequenching observed upon addition of^a^	
Transformant	Na^+^	K^+^
Nt-CPA1a	77 ± 2 (1.0)^b^	20 ± 2 (3.0)
Nt-CPA1b	24 ± 1 (2.9)	20 ± 0.3 (7.5)
Nt-CPA2	27 ± 0.1 (1.6)	27 ± 2 (5.0)
Nt-Nha	29 ± 8 (2.2)	26 ± 7 (2.9)
Nt-NhaC1	21 ± 0.3 (1.5)	29 ± 3 (2.0)
Nt-NhaC2	43 ± 0.1 (4.4)	37 ± 0.3 (7.5)
Nt-NhaC3	< 2 (0)	26 ± 1 (30.2)
Nt-NhaC4	54 ± 4.3 (0.8)	9 ± 2 (1.2)
Control	< 2 (0)	2 ± 1 (0.5)

a Vesicles from transformants expressing vector [pGEM-3Zf(+)] and antiporter genes from *N. thermophilus* were assayed under anaerobic conditions in 4 ml containing 10 mM Tris-Cl, 140 mM choline chloride, 15 mM MgCl_2_, 2.5 mM Trizma nitrate, 0.75 μM acridine orange and 500 μg protein ml^−1^. Anaerobic respiration was initiated by addition of Trizma-succinate to a final concentration of 2.5 mM. After steady-state fluorescence quenching was reached, NaCl or KCl was added to final concentrations of 3 mM for Nt-CPA1a, -CPA2, -NhaC1, -NhaC3 and -NhaC4, 5.5 mM for Nt-CPA1b and Nt-Nha, and 8.5 mM for Nt-NhaC2. KCl was added to a final concentration of 31 mM for Nt-NhaC3. All assays were adjusted to pH^37°C^ 8.5, assays for NT-NhaC2 were done at pH^37°C^ 9.5. The values presented for the subsequent per cent dequenching are from triplicate assays from two independent experiments. The percentages represent the average values of the calculated per cent dequenching and are shown with the standard error of the values.

b *K*_0.5_ values (mM) for antiporters are shown in parentheses. *K*_0.5_ values were calculated from *v* versus [S] plots exhibiting non-Michealis–Menten kinetics (Fig. 3).

Antiport activity was examined over a range of NaCl concentrations (Table 1, Fig. 3). In contrast to the Michaelis–Menten kinetics that have been reported for Na^+^(Li^+^)/H^+^ antiporters from Gram-positive bacteria (Swartz *et al*., 2007), non-linear kinetics were observed for all *N. thermophilus* antiporters (Fig. 3). Antiport activity was inhibited at high concentrations of Na^+^ (> 10 mM, substrate Na^+^-inhibition) for all proteins. Due to non-Michaelis–Menten kinetics, *K*_m_ values could not be determined. Instead, half saturation concentrations (*K*_0.5_) were determined from *v* versus [S] plots (Fig. 3). *K*_0.5_ values for the antiporter proteins varied significantly between the seven Na^+^-translocating antiporters, and ranged between 0.8 and 4.4 mM. The K^+^ optimum for the K^+^/H^+^ specific Nt-NhaC3 was 31 mM (Table 1).

**Fig. 3:**
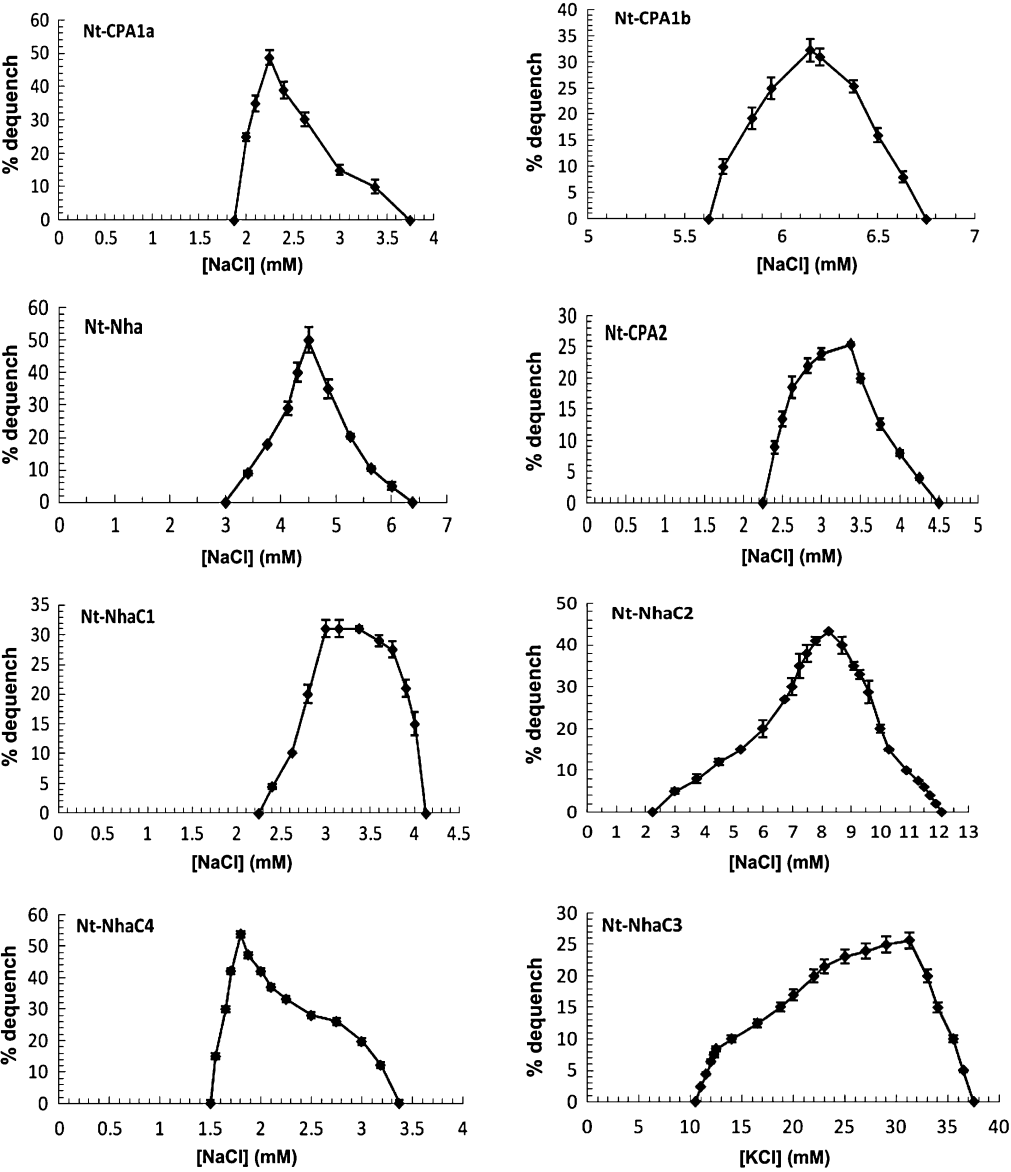
Cation/proton antiport activity of *N. thermophilus* antiporters as a function of cation concentration. Fluorescence based assays of cation/proton antiport activities of *N. thermophilus* antiporters were conducted at pH^37°C^ 8.5 (pH^37°C^ 9.5 for Nt-NhaC2) over a range of concentrations of added NaCl or KCl. The values presented for percentage dequenching are from triplicate assays from two independent vesicle preparations.

The activity profiles of *N. thermophilus* antiporters as a function of pH^37°C^ indicated alkaline pH^37°C^ optima between 8.5 and 8.8 (Table 2). Activity was greatly reduced at pH^37°C^ 7.8 and no activity was detected at pH^37°C^ 7.6 or below. The pH profile for Nt-NhaC2 is distinguished from the remaining antiporters in that it has a pH^37°C^ optimum of 9.5 and retained activity at pH^37°C^ 10.0. Higher pH values could not be tested due to decreased activity of the heterologous *E. coli* membranes used.

**Table 2:** Cation/proton antiport activity of *N. thermophilus* antiporters as a function of pH.

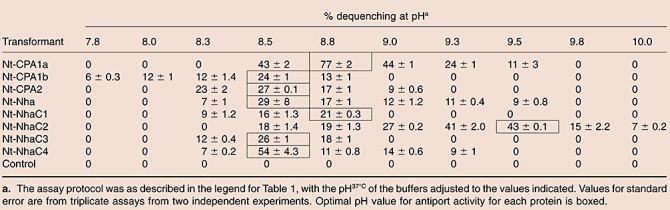

**Fig. 4:**
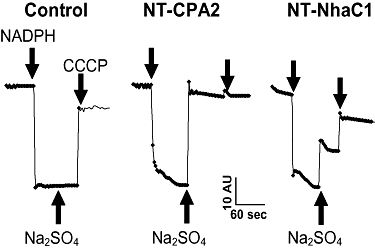
Electrogenicity of cation/proton antiport. Fluorescence-based assays of inverted membrane vesicles prepared from cells expressing the control plasmid [pGEM-3Zf(+)] and *N. thermophilus* antiporter genes were performed under anaerobic conditions as described in *Experimental procedures*. To initiate respiration, 0.6 mM of NADPH was added at the first arrow. Once the quenching reached steady state, 4 mM of NaCl (25 mM KCl for Nt-NhaC3) was added at the second arrow. The final arrow indicates addition of 10 μM carbonyl cyanide *m*-chlorophenylhydrazone. The traces shown are representative of three independent experiments. The traces for the remaining six antiporter proteins were essentially identical to that of Nt-CPA2, thus they are not shown.

Electrogenic antiport was assessed using the fluorescent probe Oxonol VI (see *Experimental procedures*). When cation/proton antiporters are used for pH homeostasis, the antiporter needs to be electrogenic rather than electroneutral (Padan *et al*., 2005). During electrogenic antiport, the inward H^+^ flux is larger than outward Na^+^ flux during a single turnover, such that net positive charge is translocated into the cell. Thus, the antiporter can be energized by Δψ (positive outside, relatively negative inside in whole cells). As a result, antiporter activity dissipates the Δψ. Electrogenic antiporter activity was observed in membrane vesicles containing each one of the eight *N. thermophilus* antiporter genes (Fig. 4). Membrane vesicles containing Nt-NhaC1 did not show a complete reversal of quenching upon addition of Na_2_SO_4_. This could be due to a different stoichiometry of Na^+^/H^+^ exchange, thus not completely consuming the Δψ. Alternatively, this particular protein may not be able to function optimally in the heterologous system used in this study.

### Expression of *N. thermophilus* antiporters is constitutive

As cation/proton antiporters play a dominant role in adaptation to alkaline stress, expression of all 12 identified antiporters was examined after adaptation of *N. thermophilus* (∼15 doubling times in continuous culture) to either an alkaline (pH^55°C^ 10.5) or relatively ‘acidic’ (pH^55°C^ 8.5) stress using quantitative reverse transcriptase PCR (qRT-PCR). Changes in the expression levels of the 12 proteins were determined in cells in mid-exponential growth phase after a shift (from optimal pH^55°C^ 9.5) to either pH^55°C^ 10.5 or pH^55°C^ 8.5 for 50 h. Samples were withdrawn from steady-state cultures of *N. thermophilus* growing continuously at the indicated pH^55°C^ value, and RNA was extracted and reverse-transcribed as described in Supporting information Experimental procedures.

**Fig. 5:**
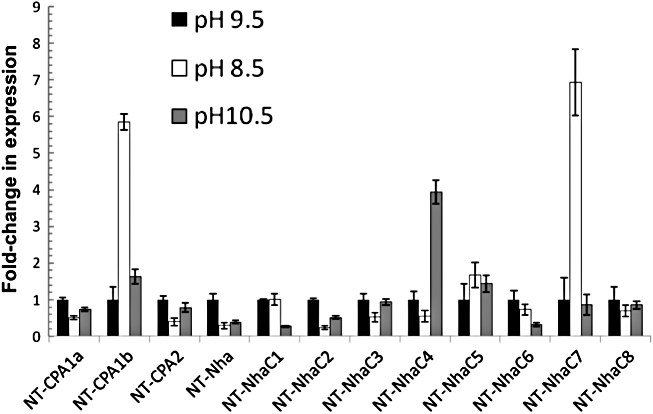
Expression of antiporter genes in *N. thermophilus* when grown at different pH^55°C^ values in continuous culture as determined by qRT-PCR. Bars indicate standard error calculated from triplicate runs from two independent experiments.

In the presence of both alkaline and ‘acid’ stress, expression of Nt-CPA1a, Nt-CPA2, Nt-NhaC1, Nt-NhaC2, Nt-NhaC3, Nt-NhaC5, Nt-NhaC6 and Nt-NhaC8 remained constitutive (Fig. 5). Consistent with its relatively low-pH minimum for activity (Table 2), expression of the Na^+^(K^+^)/H^+^ antiporter Nt-CPA1b showed a sixfold increase after adaptation to pH^55°C^ 8.5. Nt-NhaC4 showed a fourfold increase in expression after acclimation to pH^55°C^ 10.5 (Fig. 5). Interestingly, Nt-NhaC2 showed a slight decrease in expression at pH^55°C^ 10.5 even though it had the most alkaline pH optimum when assayed in inverted membrane vesicles. Nt-NhaC7 showed a sevenfold increase in expression at pH^55°C^ 8.5. This protein did not show any antiport activity in inverted membrane vesicles nor did it complement the Na^+^-sensitive phenotype of *E. coli* KNabc. However, it did complement the K^+^-uptake deficiency of *E. coli* TK2420 indicating that it has K^+^-transport capability.

## Discussion

The poly extremophile *N. thermophilus* is a prototype for robust growth at combined extremes of pH, temperature and salt concentration. The mechanism(s) responsible for this extraordinary feat were the focus of this study. *N. thermophilus* exhibited unusual energy-dependent and -independent ΔpH homeostasis over the pH^55°C^ range for growth, a characteristic not observed in the alkalithermophiles studied (Cook *et al*., 1996; Olsson *et al*., 2003). At high pH^55°C^ values (i.e. > 9.5), growth decreased and the intracellular pH increased to values > 8.8. Despite poor growth at these high pH^55°C^ values, the cells continued to maintain a cytoplasmic pH that was more acidic than the external milieu. The ability of some bacteria to maintain a ΔpH that is energy-independent has been attributed to the buffering capacity of the cytoplasm to sequester or release protons (Booth, 1985). The buffering capacity of the cytoplasm is due to the presence of titratable groups (e.g. basic and acidic amino acids, both free and attached to proteins). Analysis of the proteome of *N. thermophilus* showed that, consistent with being a halophilic microorganism, the isoelectric point of proteins is predominantly acidic, ranging between 4 and 5 (Fig. S3). Thus, at the alkaline intracellular pH of *N. thermophilus*, a large amount of proteins will be negatively charged. As the internal pH increases, the charge will become more negative and therefore, to reach the demand by the cell for overall neutrality on both sides of the membrane, cations, including protons may enter the cell. This leads to a more acidic interior. It is important to note that when incubated at pH^55°C^ values greater than 10.3, cells of *N. thermophilus* grow with extended doubling times (15 versus 3.5 h at optimal pH), do not reach OD_600_ values greater than 0.1, and quickly lose viability after reaching stationary phase. The intracellular pH under these conditions increases beyond 9.5. Cessation of growth could be due to saturation of the intracellular buffering capacity and alkalinization of the cytoplasm.

Intracellular K^+^, which is tightly regulated at 200–250 mM during growth at optimal conditions, sharply increases to almost 550 mM at extracellular pH^55°C^ 10.5; and intracellular Na^+^ increases fourfold from 8 to 33 mM. We hypothesize that this sudden increase in K^+^ serves to protect intracellular proteins from the cytotoxic effects of Na^+^. Alternatively, the increase in intracellular K^+^ (and Na^+^) may act to neutralize the net negative charge of the cytoplasm.

Alkaliphiles must maintain a cytoplasm pH that is compatible with the functional and structural integrity of cytoplasm proteins supporting growth. Alkaliphiles generally acidify the cytoplasm, and maintain an intracellular pH that is moderately alkaline (pH 7.5–8.5) (Krulwich *et al*., 1996; Padan *et al*., 2005). Among the strategies used for cytoplasm acidification, increased activity of monovalent cation proton antiporters plays a dominant role in cytoplasm pH homeostasis (Padan *et al*., 2005). We identified 12 putative antiporters in the genome of *N. thermophilus*. Of these 12 homologues, 8 of them showed strong sequence identity to the Na^+^/H^+^ antiporter NhaC. The four remaining genes had homology to members of the monovalent cation/proton antiporter-1 and cation/proton antiporter-2 families. All these protein families contain transporters that play roles in cytoplasmic pH regulation, extrusion of intracellular Na^+^ and cell volume regulation (Waser *et al*., 1992; Southworth *et al*., 2001; Radchenko *et al*., 2006; Wei *et al*., 2007).

Eight of the 12 *N. thermophilus* antiporter homologues displayed antiport activity in membrane vesicles prepared from *E. coli* KNabc. The antiport capacities of the proteins support their ability to play a major role in cytoplasm acidification in *N. thermophilus*. Electrogenicity is an important property for Na^+^/H^+^ antiporters that support alkali resistance (Padan *et al*., 2005), and all *N. thermophilus* antiporters showed Na^+^ and K^+^-dependent consumption of the Δψ. Seven antiporter proteins exhibited strong Na^+^(K^+^)/H^+^ antiport activity, and one showed only K^+^/H^+^ antiport activity. Together, the eight antiporters functioned over a range of Na^+^ and K^+^ concentrations, consistent with the ability of this bacterium to grow over a range of salinities. The ability to use K^+^/H^+^ antiport in addition to Na^+^/H^+^ antiport for cytoplasm acidification is beneficial for an anaerobic halophilic alkalithermophile. Aerobic microorganisms typically use H^+^-coupled bioenergetics; there is no competition for the intracellular Na^+^ substrate. On the other hand, the anaerobic alkalithermophiles studied to date have Na^+^-coupled mechanisms for pH homeostasis and solute transport (Speelmans *et al*., 1993; Prowe *et al*., 1996; Ferguson *et al*., 2006). As a result, the Na^+^-coupled ATPase will compete with the Na^+^/H^+^ antiporters for intracellular Na^+^. In this case, K^+^/H^+^ antiporters can continue to acidify the cytoplasm after Na^+^/H^+^ antiporters sufficiently reduce the cytoplasmic Na^+^ content.

The overlapping pH profiles of *N. thermophilus* antiporters further supports their ability to play a role in mediating concomitant contributions to intracellular pH and/or salt tolerance of *N. thermophilus.* Optimal antiport activity in inverted membrane vesicles was observed within the pH^37°C^ range of 8.5–8.8, similar to the intracellular pH in energized cells of *N. thermophilus.* This indicates that the antiporters are biochemically compatible with the intracellular pH of *N. thermophilus*. Furthermore, constitutive expression of the majority of antiporter genes under both acidic and alkaline stress implies that their products make synergistic contributions to pH and/or salt tolerance of *N. thermophilus* and a ‘ready’ mechanism to combat sudden changes in the external environment. Expression of *nt-CPA1b, nt-NhaC4* and *nt-NhaC7* were significantly altered under stress conditions indicating pH-dependent regulation of these proteins. It is possible that these proteins in particular play major adaptive roles under the respective stress conditions.

**Fig. 6:**
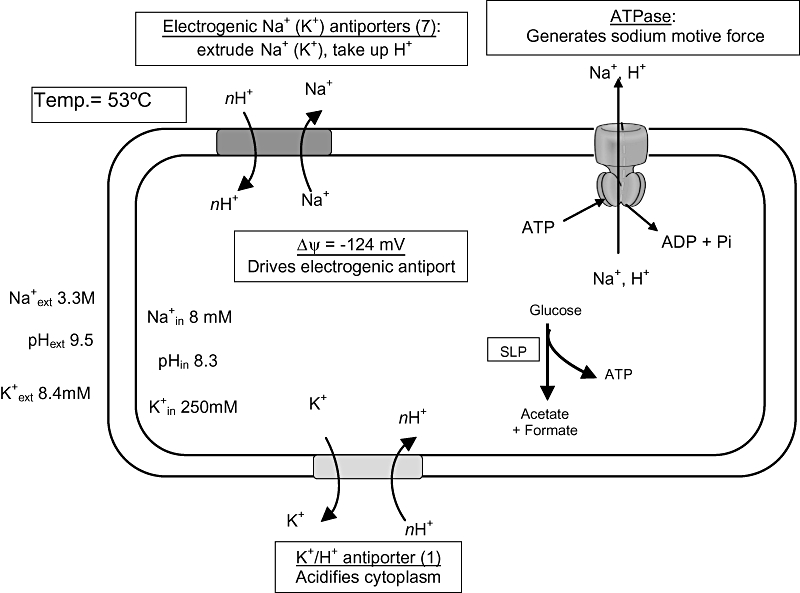
Schematic diagram of bioenergetic processes in *N. thermophilus*. SLP, substrate level phosphorylation.

A model summarizing the bioenergetic processes presented and discussed in *N. thermophilus* is shown in Fig. 6. *N. thermophilus* is a fermentative bacterium that generates the bulk of its ATP via substrate level phosphorylation producing acetate and formate. The membrane-bound F-type ATPase plays an important role in establishing the electrochemical gradient of Na^+^ ions, fuelled by ATP that is rapidly produced from substrate level phosphorylation. The result of sodium pumping also leads to the formation of a significant Δψ (−124 mV), which contributes to the overall electrochemical gradient of Na^+^ ions. This is vitally important because the cation/proton antiporters in *N. thermophilus* are coupled to Δψ. *N. thermophilus* utilizes two distinct mechanisms for cytoplasm acidification under conditions of high salt concentration and elevated temperature. At extracellular pH^55°C^ values at and below the optimum, acidification of the cytoplasm is achieved via a large cohort of electrogenic cation/proton antiporters that are able to translocate Na^+^ and K^+^ ions in exchange for protons. As the extracellular pH^55°C^ increases, energy-dependent antiport activity stops, and acidification is probably achieved by physiochemical forces such as cytoplasmic buffering. These strategies allow *N. thermophilus* to adapt to fluctuations in extracellular pH and Na^+^ ion concentration under conditions where the availability of energy generating substrates varies.

## Experimental procedures

### Bacterial strains and culture conditions

Strains used in this study are shown in Table S2. *N. thermophilus* strain JW/NM-WN-LF^T^ (= DSM 18059^T^ = ATCC BAA-1301^T^) was grown anaerobically at 53°C in carbonate-buffered medium as described previously (Mesbah *et al*., 2007). *E. coli* KNabc (Δ*nhaA*Δ*nhaB*Δ*chaA*) was grown routinely in LBK medium, pH 7.5 (Goldberg *et al*., 1987). *E. coli* JM109 was used for routine cloning procedures and was grown in LB medium. When antibiotics were added to the medium for selection or plasmid maintenance, concentrations used were 100 μg ampicillin ml^−1^, 50 μg kanamycin ml^−1^ and 50 μg erythromycin ml^−1^.

### Determination of bioenergetic parameters

For determination of ΔpH, ΔΨ and *pmf*, *N. thermophilus* was grown in batch culture at pH^55°C^ 9.5, 53°C in the presence of 3.3 M Na^+^. The superscript preceding the pH value indicates the temperature at which the pH meter was calibrated and pH measured as described (Wiegel, 1998; Mesbah and Wiegel, 2006).

Cells were harvested during mid-exponential phase by centrifugation (6000 *g*, 30 min, 24°C) and washed three times in sterile anaerobic carbonate-buffered medium, pH^55°C^ 9.5. All washing steps were carried out anaerobically inside a Coy anaerobic chamber (Coy Laboratory Products, Grass Lake, Michigan). Cells were resuspended to a final OD_600_ of 1.0 in anaerobic carbonate-buffered medium adjusted to the pH^55°C^ being studied. These cell suspensions (non-growing) were energized by the addition of sucrose (0.5% wt/vol.) and incubation at 53°C for 20 min. Energization caused an increase in gas pressure at all tested pH values, indicating that the cells remained active during the assay period (subculturing and dilution to extinction further confirmed viability of the cells). The final pH^55°C^ values were determined, then energized cell suspensions (2.0 ml) were then transferred by syringe to sealed serum vials (10 ml capacity, nitrogen in gas phase) containing one of the following isotopes: [^14^C]methylamine (5.4 μM) or [^3^H]tetraphenylphosphonium^+^ (TPP^+^, 1 μM). After incubation for 5 min at 53°C, 0.9 ml of the culture were rapidly centrifuged through 300 μl of silicone oil (13 000 *g*, 3 min, 24°C). Twenty microlitres of supernatant were removed. The tubes with remaining contents were frozen at −80°C for at least 2 h. The bottoms of the tubes containing cell pellets were removed with dog-nail clippers; the supernatant and cell pellets were separately dissolved in scintillation fluid, and radioactivity (cpm) was determined with an LKB Wallac 1214 Rack-Beta scintillation counter.

Intracellular volume (5.6 ± 0.34 μl mg^−1^ protein) was determined from the difference in partitioning of [^3^H]water (1 mM) and [^14^C]polyethylene glycol. Polyethylene glycol is not metabolized by *N. thermophilus*. The membrane potential across the membrane was calculated from the uptake of [^3^H]TPP^+^ according to the Nernst relationship. Non-specific TPP^+^ binding was estimated from valinomycin- and nigericin-treated cells (10 μM each). These inhibitors cause complete growth-arrest when added to cultures of *N. thermophilus* during the exponential growth phase, thus they are membrane active with this bacterium. The ΔpH was determined from the distribution of [^14^C]methylamine with the Henderson–Hasselbach equation, and ZΔpH was calculated by 59 mV multiplied by the ΔpH. The phosphorylation potential, ΔGp, was calculated with the equation: ΔG_p_ = ΔG° + 2.3RT log[ATP]/[ADP][P_i_]. The value used for ΔG° was 33.3 kJ mol^−1^, or the equivalent of −347 mV (Nicholls and Ferguson, 2002). Intracellular concentrations of ATP, ADP and Pi were determined as described (Olsson *et al*., 2003).

### Measurement of intracellular sodium and potassium ion concentrations

Intracellular Na^+^ and K^+^ concentrations were measured as described (Olsson *et al*., 2003). Details are explained in Supporting information Experimental procedures.

### DNA extraction, cloning and plasmids

PCR was carried out on *N. thermophilus* genomic DNA using Phusion™ High-Fidelity DNA polymerase (New England BioLabs, Ipswich, MA). PCR primers used are listed in Table S2. All genes were cloned with their native Shine–Dalgarno sequences behind the T7 promoter in pGEM-3Z(f+). Basal levels of expression of the cloned genes, without addition of inducer, were used for all experiments. Complete DNA sequencing was used to ensure that the plasmids ultimately used were free of errors. Details of genome sequencing, identification of putative transporter genes, PCR reactions and plasmid constructions are provided in Supporting information Experimental procedures.

### Complementation assays in *E. coli* Knabc

All complementation assays were done under anaerobic conditions. Recombinant plasmids were transformed into Na^+^(K^+^)(Ca^2+^)/H^+^ antiporter-deficient *E. coli* KNabc. The plasmid pGEM-3Zf(+) was used as a negative control. For studies of complementation of the Na^+^- and alkali-sensitive phenotypes of *E. coli* KNabc, the test and control transformants were cultured anaerobically overnight in LBK medium, pH^37°C^ 7.5. Two hundred microlitres of the overnight grown cultures was transferred into 5 ml of LBK adjusted to different pH values with different NaCl concentrations. Cell growth was monitored by measuring optical density at 600 nm.

### Preparation of inverted membrane vesicles

Inverted membrane vesicles were prepared as described previously (Rosen, 1986). Details of buffers used are described in Supporting information Experimental procedures.

### Assays of ΔpH-dependent antiport activity in inverted membrane vesicles

Antiport assays were conducted anaerobically in 10 mM Tris-Cl, 140 mM choline chloride, 15 mM MgCl_2_, 2.5 mM Trizma nitrate, 0.75 μM acridine orange and 500 μg protein ml^−1^. The pH^37°C^ was adjusted to values between 7.5 and 10.0. Measurements were conducted using a Turner Designs TD-700 laboratory fluorometer with peak excitation 486 nm and peak emission 510 nm. Respiration was initiated by addition of Trizma-succinate to a final concentration of 2.5 mM. The chloride content of the buffer was high enough to ensure that the *pmf* established upon addition of an electron donor was entirely due to a ΔpH, acidic inside the inverted membrane vesicles (Rosen and Futai, 1980). Establishment of the ΔpH was monitored by quenching of the fluorescence of acridine orange. Cation addition results in dequenching of fluorescence that reflects cation-dependent proton movement out of the inverted membrane vesicles. At the end of the assay, 12 mM of ammonium chloride was added to dissipate the remaining *pmf* and bring fluorescence back to baseline.

### Fluorescence-based assays of ΔΨ generation and antiporter dependent consumption

ΔΨ-dependent fluorescence of oxonol VI was used to measure the generation of a membrane potential, positive inside, with the addition of 0.6 mM of tetra(cyclohexylammonium)-NADPH. Electrogenicity of antiporter genes was evaluated by adding 4 mM Na_2_SO_4_ to energized membranes and observing a reversal of the quench. Reversal of quenching represents antiporter-dependent consumption of ΔΨ, which reflects activity of an electrogenic antiporter (Padan *et al*., 2005). Fluorescence was brought back to baseline by adding of 10 μM of carbonyl cyanide *m*-chlorophenylhydrazone, a protonophore that abolishes the ΔΨ. The assay mixture contained 10 mM bis-[tris(hydroxymethyl)methylamino]-propane, 5 mM MgSO_4_, 200 mM K_2_SO_4_, 1 μM nigericin and 1 μM oxonol VI (pH^37°C^ 8.0–9.5). Measurements were made on a Turner Designs TD-700 laboratory fluorometer. The peak excitation and emission wavelengths were 523 nM and 630 nm respectively. Final concentration of vesicle protein was 500 μg ml^−1^.

### Continuous culture, RNA isolation and qRT-PCR

For continuous culture experiments, *N. thermophilus* was grown at 53°C with continuous stirring in a custom-made glass fermentor with a working volume of 500 ml. The same carbonate-buffered medium used for the batch cultures was used; the medium was kept anaerobic by a continuous flow of sterile N_2_. The continuous culture system was operated and maintained at a constant pH^55°C^ by the adjustment of the medium in the reservoir to the target pH^55°C^. Medium pH^55°C^ was adjusted by addition of either 10 N HCl, 3 M Na_2_CO_3_ or 5 M NaOH. The pH^55°C^ in the fermentor was measured with an autoclavable glass electrode (Femprobe®, Broadley James Corp., CA). The pH^55°C^ values detected by the immersed electrode were checked for accuracy against an external electrode calibrated at 55°C with appropriately preheated buffers (Wiegel, 1998; Mesbah and Wiegel, 2006). The cultures were maintained at steady-state at OD_600_ in the range of 0.20–0.22 for at least 50 h (equivalent to approximately 15 doubling times). Continuous culture runs were conducted in duplicate for each culture condition tested.

Samples for RNA extraction were withdrawn from steady-state cultures of *N. thermophilus* growing at the indicated pH^55°C^ value. Details of RNA extraction, reverse transcription, and qPCR are provided in Supporting information Experimental procedures.

Differences in the transcript levels of the antiporter genes in response to different extracellular pH^55°C^ values were calculated according to the model proposed by Pfaffl (2001), using the *recA* gene as a reference gene.

## Note added in proof

The GenBank accession number for the genome sequence of *N. thermophilus* is NC_010718.
