# Supplementary material for: The halophilic alkalithermophile Natranaerobius thermophilus adapts to multiple environmental extremes using a large repertoire of Na+(K+)/H+ antiporters
Source: Mol Microbiol. 2009 Aug 28;74(2):270–81. doi: 10.1111/j.1365-2958.2009.06845.x (PMC2764116; doi:10.1111/j.1365-2958.2009.06845.x)
Supplement: Supplementary file 1 [file mmi0074-0270-SD1.pdf]

Supporting Information

Experimental Procedures

Genome sequencing and identification of transporter protein genes. The genome of *Natranaerobius thermophilus* strain JW/NM-WN-LF^{T} was sequenced by the random shot-gun method (see www.jgi.doe.gov for details of library construction and sequencing). Complete predicted protein sequences were searched against a curated set of proteins with family assignment for similarity to known or putative transporter proteins and against a nonredundant general protein database using a semiautomated pipeline (Ren *et al.*, 2007). Manual annotation for final assignments was based on the number of hits to the transporter database, maximum, minimum, and average BLAST E values and cluster of orthologous group (COG) assignments. The annotated antiporter genes used in this study are shown in Table S1.

Measurement of intracellular sodium and potassium ion concentrations. Cells were harvested from pH$^{55°C}$ controlled batch culture, and resuspended in isotonic anaerobic medium adjusted to the pH$^{55°C}$ value being studied. This cell suspension was then centrifuged at 13,000 x *g* for 1 min at 24ºC. Five hundred microliters of supernatant was removed, and cell pellets were digested with 3N HNO_3 for 24 hours at room temperature. Sodium and potassium concentrations in cell digests and supernatants were analysed by flame photometry. Corrections were made for extracellular contamination of the cell pellet by Na^+ and K^+ as described (Olsson *et al.*, 2003).

Preparation of inverted membrane vesicles. Inverted membrane vesicles were prepared from *E. coli* KNabc transformants in late-exponential growth phase. For cation/proton antiport assays, cell pellets from 1.5 L of culture (grown anaerobically for 24 hrs) were washed 3 times with 30

mL of buffer containing 10 mM Tris-chloride (pH$^{37°C}$ 8.0), 140 mM choline chloride, 0.5 mM dithiothreitol and 10% (vol/vol) glycerol. Cells were then resuspended in 20 mL of membrane buffer (100 mM Tris-chloride [pH$^{37°C}$ 8.0], 140 mM choline chloride, 15 mM $MgCl_2$, 0.5 mM dithiothreitol, 1 mM phenylmethylsulfonyl fluoride [PMSF], and 10% glycerol [vol/vol]). DNaseI (2 mg) was added and the cell suspension was shaken briefly. The following steps were performed at 4ºC: cell suspensions were disrupted by passage three times through a pre-cooled French pressure cell at 10,000 psi. Unbroken cell material was removed by two low speed centrifugations (8,000 x *g* for 10 mins), and the membranes were collected by ultracentrifugation at 250,000 x *g* for 1 hour. Membranes were washed in membrane buffer and centrifuged as described above. Washed membranes were resuspended in 1.5 mL of membrane buffer, and then were shock frozen in a slurry of dry ice and 100% ethanol and stored at -80ºC. Vesicles used in assays of membrane potential ($\Delta\psi$) generation were prepared in a similar way, but were washed and resuspended in membrane buffer composed of 10 mM bis-[tris(hydroxymethyl)-methylamino]-propane (BTP), pH$^{37°C}$ 8.0, 10% glycerol and 1 mM PMSF. The final concentration was 30-50 mg protein/mL. Protein concentrations were determined by the Bicinchoninic acid (BCA) assay using bovine serum albumin as standard.

DNA extraction, cloning and plasmids. High molecular weight genomic DNA was isolated from *N. thermophilus* cells by the SDS/phenol/chloroform extraction method as described (Wilson, 1997). The 12 predicted antiporter genes and their native Shine-Dalgarno sequences were cloned behind the T7 promoter of pGEM-3Zf(+). The PCR primers listed in Table S2 were used to amplify the genes from *N. thermophilus* genomic DNA. The PCR reactions used 1 unit of Phusion™ High-Fidelity DNA polymerase (New England Biolabs), 0.5 µM each of forward and

reverse primer and ~ 50 ng of genomic DNA. The PCR cycling conditions consisted of an initial denaturation at 98ºC for 30 secs, followed by 25 cycles of: denaturation (98ºC for 10 secs), annealing (66ºC for 30 secs for *nt-NhaC1, nt-NhaC7, nt-NhaC8* and *nt-NhaC4*, 62ºC for 30 secs for remaining 8 genes), extension (72ºC for 45 secs), then a final extension at 72ºC for 5 minutes. The PCR products were purified, digested with *Bam*HI and *Xba*I *(nt-NhaC8* was digested with *Bam*HI and *Sal*I), and ligated into *Bam*HI- and *Xba*I (*Sal*1)-digested pGEM-3Zf(+) using T4 DNA Ligase (New England Biolabs). For all plasmid selections, blue-white screenings in *E.coli* JM109 were performed and complete DNA sequencing was used to ensure that the plasmids ultimately used were free of errors. The plasmids were transformed into *E. coli* KNabc by electroporation.

RNA isolation and qRT-PCR. Cells were harvested by rapid centrifugation at 4°C, then lysed by resuspension in 1 mL of TRIZOL® reagent (Invitrogen Inc., Carlsbad CA) and incubation at room temperature for 10 min. Chloroform, 0.2 mL was then added and the lysate was incubated for a further 5 min at room temperature. Aqueous and organic phases were separated by centrifugation, (10,000 rpm, 4°C, 15 min). Total RNA was precipitated from the aqueous phase according to manufacturer's instructions. RNA pellets were washed 3 times in 75% ethanol and then dissolved in DEPC-treated water. Final RNA concentrations were 200-250 ng/µL.

Prior to reverse-transcription, total RNA was treated with RNase-free DNase I (amplification grade, Invitrogen). Two independent reverse-transcriptase (RT) reactions were performed for each individual sample (SuperScript™ III, Invitrogen). Reverse transcriptase reactions were primed with random hexamers.

Quantitative PCR was performed in 25 µL reaction mixtures containing 100 ng cDNA

(corresponding to 100 ng starting RNA), 200 – 700 nM of each primer (Table S3), and 12.5 µL of SYBR®GReenER™ qPCR supermix (Invitrogen). Primers were designed using the OligoPerfect™ Designer program (http://www.tools.invitrogen.com). Samples were run in triplicate on an iCycler (Bio-Rad) with the following conditions: 10 min at 95ºC and 40 cycles of 95ºC for 15 s and 60ºC for 60 s, followed by melt-curve analysis. An eight-point standard curve was run in parallel for each primer set to assess primer efficiency. A no-template-control (NTC) and – reverse transcriptase (-RT) control were run for each RNA sample to ensure absence of genomic DNA contamination. Primer amplification efficiencies were all within 5% of each other (Table S3).

Table S1. List of predicted antiporter proteins from *N. thermophilus*

Gene designation	Open reading frame designation	Protein family	Closest homolog (GeneBank designation)	% Amino acids overlap	Reported antiport activity
Nt-CPA1a	nther1135	CPA1	Na^+/H^+ exchanger *Alkaliphilus metalliredigens* QYMF (YP_001322502)	42	n.d.
Nt-CPA1b	nther0276	CPA1	Na^+/H^+ exchanger *Alkaliphilus oremlandii* OhILAs (YP_001513623)	38	n.d.
Nt-CPA2	nther0270	CPA2	Na^+/H^+ exchanger *Alkaliphilus metalliredigens* QYMF (YP_001322136)	48	n.d.
Nt-Nha	nther0107	CPA3	Na^+/H^+ antiporter ShaA *Clostridium tetani* E88 (NP_781965)	35	n.d.
Nt-NhaC1	nther2649	NhaC	Na^+/H^+ antiporter *Chromohalobacter salexigens* DSM 3043 (YP_575253)	56	n.d.
Nt-NhaC2	nther2454	NhaC	Na^+/H^+ antiporter *Fusobacterium nucleatum* subsp *nucleatum* ATCC 25586 (NP_604314)	51	n.d.
Nt-NhaC3	nther2242	NhaC	Na^+/H^+ antiporter	55	n.d.

			Fusobacterium nucleatum subsp. *polymorphum* ATCC 10953 (ZP_02240864)		
Nt-NhaC4	nther1834	NhaC	Na^+/H^+ antiporter *Bacillus pseudofirmus* OF4 (AAC45432)	36	Yes (Ito *et al.*, 1997)
Nt-NhaC5	nther0736	NhaC	Na^+/H^+ antiporter *Alkaliphilus metalliredigens* QYMF (YP_001322347)	44	n.d.
Nt-NhaC6	nther0921	NhaC	Na^+/H^+ antiporter *Vibrio parahaemolyticus* (NP_796997)	40	No (Radchenko *et al.*, 2006)
Nt-NhaC7	nther2636	NhaC	Na^+/H^+ antiporter *Alkaliphilus metalliredigens* QYMF (YP_001321248)	44	n.d.
Nt-NhaC8	nther2651	NhaC	Malate:$2H^+$/ Lactate:Na^+ antiporter YqkI *Bacillus subtilis* (ZP_00739085)	63	Yes (Wei *et al.*, 2000)

n.d.; not determined

Table S2. Bacterial strains, plasmids and oligonucleotides

Strain, plasmid, or oligonucleotide	Relevant characteristics or sequence[a]	Source or reference
Natranaerobius thermophilus JW/NM-WN-LF^T DSM 18059^T ATCC BAA-1301^T	Halophilic, alkalithermophilic, anaerobic, Firmicute	(Mesbah *et al.*, 2007)
	<u>*E. coli* strains</u>	
KNabc	Δ*nhaA* Δ*nhaB* Δ*chaA*	(Nozaki *et al.*, 1996)
JM109	*end*A1, *rec*A1, *gyr*A96, *thi*, *hsd*R17 (r_k^-, m_k^+), *rel*A1, *sup*E44, Δ(*lac*-*pro*AB), [F´ *tra*D36, *pro*AB, *laq*I^{q}ZΔM15].	Promega
<u>Plasmids</u>		
pGEM-3Zf(+)	Cloning vector, Ap^r	Promega
pNMM1	*nt-CPA1a* (1.2 kb) PCR product (primers NT476/ BF1 and NT476/ XR1) in BamHI/ XbaI digested pGEM-3Zf(+)	This study
pNMM2	*nt-CPA1b* (1.2 kb) PCR product (primers NT1314/ BF1 and NT1314/ XR1) in BamHI/ XbaI digested pGEM-3Zf(+)	This study
pNMM3	*nt-CPA2* (1.2 kb) PCR product (primers NT1320/ BF1 and NT1320/ XR1) in BamHI/ XbaI digested pGEM-3Zf(+)	This study
pNMM4	*nt-Nha* (1.5 kb) PCR product (primers NT1499/ BF1 and NT1499/ XR1) in BamHI/ XbaI digested pGEM-3Zf(+)	This study
pNMM5	*nt-NhaC1* (1.3 kb) PCR product (primer NT1898/ BF1 and NT1898/ XR1) in BamHI/ XbaI digested pGEM-3Zf(+)	This study
pNMM6	*nt-NhaC2* (1.3 kb) PCR product (primer NT2084/ BF1 and NT2084/ XR1) in BamHI/ XbaI digested pGEM-3Zf(+)	This study

pNMM7	*nt-NhaC3* (1.4 kb) PCR product (primer NT2291/ BF1 and NT2291/ XR1) in BamHI/ XbaI digested pGEM-3Zf(+)	This study
pNMM8	*nt-NhaC4* (1.4 kb) PCR product (primer NT2693/ BF1 and NT2693/ XR1) in BamHI/ XbaI digested pGEM-3Zf(+)	This study
pNMM9	*nt-NhaC5* (1.4 kb) PCR product (primer NT855/ BF1 and NT855/ XR1) in BamHI/ XbaI digested pGEM-3Zf(+)	This study
pNMM10	*nt-NhaC6* (1.5 kb) PCR product (primer NT679/ BF1 and NT679/ XR1) in BamHI/ XbaI digested pGEM-3Zf(+)	This study
pNMM11	*nt-NhaC7* (1.4 kb) PCR product (primer NT1896/ BF1 and NT1896/ XR1) in BamHI/ XbaI digested pGEM-3Zf(+)	This study
pNMM12	*nt-NhaC8* (1.5 kb) PCR product (primer NT1910/ BF1 and NT1910/ SR1) in BamHI/ XbaI digested pGEM-3Zf(+)	This study
Oligonucleotides		
NT476/ BF1	CGGCGGATCCGGATTGAGGGGATTTAACTAATGTTGG	This study
NT476/ XR1	GGGGTCTAGACGTTACTCCTCTATGCAATCACAATT	This study
NT1314/ BF1	CCCCGGATCCCCTAGAGGTGACCTTAATTGACAATC	This study
NT1314/ XR1	GGGCTCTAGAGGTTACACCTCCTTAGTTGACG	This study
NT1320/ BF1	CCCCGGATCCGGTTTTTAAGAAAGGGATGATTTACAATG	This study
NT1320/ XR1	GGGCTCTAGAGGTTATTCTATTGAGGAAAGTTCAGCAGT	This study
NT1499/ BF1	GGCCGGATCCCCTAGGAGGTTTTAGTAGTGTCTTTT	This study
NT1499/ XR1	GGGCTCTAGAGGCTAGAAAGGATTACCATT	This study

NT1898/ BF1	CCCGGGATCCCCAGGAAGGAAAAGGAGGTGAAACTA	This study
NT1898/ XR1	CCCCTCTAGACCTTATAGCCGGGAGTAACCCC	This study
NT2084/ BF1	CCCCGGATCCTTATGGATCAAAAAGAAATTAAACCG	This study
NT2084/ XR1	CCCCTCTAGACCCTAAGCGCTATCTTCTGAAGGTTT	This study
NT2291/ BF1	CCCGGGATCCCCATGAAAAATGAAATTGACAAGGCT	This study
NT2291/ XR1	GGGCTCTAGAGGTTACCCTTCAATAGATTCTTCTTGTTC	This study
NT2693/ BF1	CCCCGGATCCCCATAGAAGGAGGTTTCAATATGAAT	This study
NT2693/ XR1	GGGCTCTAGAGGTTAATGTCTCTCCAGTTTCTTACC	This study
NT855/ BF1	CCGCGGATCCCCATGAACGAAAGTAATCAGAATTTT	This study
NT855/ XR1	CCCCTCTAGACCTTAAGCCTCTGTTTTCTCATAT	This study
NT679/ BF1	CCGCGGATCCCCAAGGAGGACATACACATGGAACAT	This study
NT679/ XR1	CCCCTCTAGACCTTAATAATTTAATTTACTGGATTCATT	This study
NT1896/ BF1	GGGCGGATCCGGAAAGGGGGGATTTTAATGGGTGAA	This study
NT1896/ XR1	CCCCTCTAGACCTTAATTGTCAGCTTCTTCAGTCTT	This study
NT1910/ BF1	CCCGGGATCCGGAGGAGGGATCTCTTATTGTCTGAC	This study

NT1910/ SR1	GGGGGTCGACGGTTAATCACCAGCTATTCCGGC	This study

[a] Ap^r, ampicillin resistance; oligonucleotide sequences are given 5’ to 3’; restriction sites are underlined

Table S3. Oligonucleotides used in qPCR, product sizes, optimal primer concentrations used and PCR efficiency

Primer name	Sequence (5’ → 3’)	Product Size (bp)	Conc. used in qPCR (nM)	PCR Efficiency (%)
RecA-F	TTCATGCGGTAGCAGAAGCTCAGA	141	200	102.3
RecA-R	TGCTCGCCTGTATCTGGTTGTGAA		200	
NTCPA1a-F	ATATCCTAGCACGACTTGCGGGAA	141	200	104.5
NTCPA1a -R	AAATGGCTAGACCGATTGCTACGC		200	
NTCPA1b-F	GCCGGAGCTGAACTACACTT	119	600	102.9
NTCPA1b -R	TGTGACCATCTGGCACCTAA		600	
NTCPA2-F	TGAAATGGCCGACAGTGATA	92	200	105.3
NTCPA2-R	GTAGGCCGGCAACTTGTAAA		200	
NTNha-F	GTAACAGATGCCGGTGGACT	244	200	104.2
NTNha -R	GAGCCCCTTGCATTACACAT		200	
NTNhaC3-F	GTAACAGATGCCGGTGGACT	244	500	105.5
NTNhaC3-R	GAGCCCCTTGCATTACACAT		500	
NTNhaC2-F	GTTTGGTATTGGCTGGGCTA	168	200	106.7
NTNhaC2-R	AAAAGGCCAGTCCACCTTCT		200	
NTNhaC1-F	ACTTGCTATTGCATTGACCCTGGC	175	600	104.6
NTNhaC1-R	AGTCAGCACTGTAGCATTCCACCT		600	
NT-NhaC4-F	TTAGGAGTAGGTGGAGGACTTGGT	143	200	107.1
NTNhaC4-R	AGGAGCTGTCACCCACAACATTGA		200	
NTNhaC5-F	ATGAGAGTTCCCCCGATACC	86	200	103.7
NTNhaC5-R	GCGAATGAAGATCCCTGAAA		200	
NTNhaC6-F	TGGGCCTATTTTTGCAACTC	118	500	102.0
NTNhaC6-R	TCCCCAACCAACTATGGGTA		500	
NTNhaC7-F	GCTGGCTCTATCAGGAATCG	138	200	106.7
NTNhaC7-R	CCGGCTGTACTTGAGGCTAC		200	
NTNhaC8-F	ATGTAGGAACGGCACCAGAG	241	700	103.7

NTNhaC8-R	GCGAATCCATCATAGGCAGT		700	

Figure S1. Effect of inhibitors on intracellular $pH^{55°C}$ in cell suspensions of *N. thermophilus*. A) Effect of inhibitors on intracellular pH in energized cell suspensions at **pH 9.5**. B) Effect of inhibitors on intracellular pH in energized (grey) and non-energized (white) suspensions of *N. thermophilus* at **pH 10.5**. TCS: 3,3',4',5-tetrachlorosalicylanilide; CCCP: carbonyl cyanide *m*-chlorophenylhydrazone. Bars indicate standard error from three replicate experiments. Intracellular pH was determined as described in Experimental Procedures, inhibitors were added to a final concentration of 10 µM. CCCP was added to a final concentration of 5 µM.

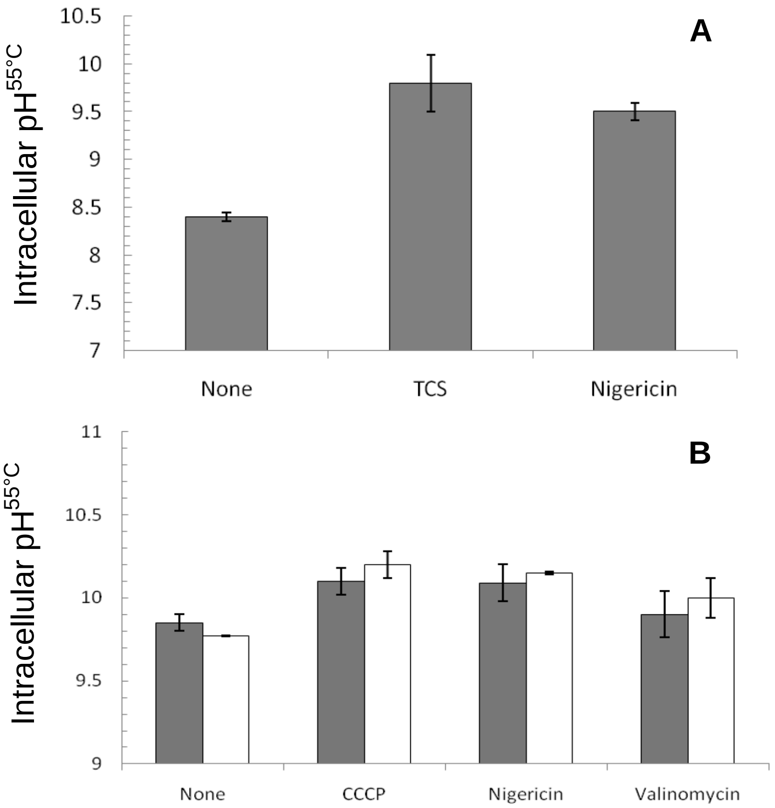

Figure S2. Effect of K^+ concentration on the growth of *E. coli* TK2420 transformants of *N. thermophilus* antiporters. Transformants with vector control pGEM-3Zf(+), or expressing *N. thermophilus* antiporter genes, were grown anaerobically on minimal medium as described by Epstein and Kim (1971), $pH^{37°C}$ 7.5, containing indicated concentrations of K^+.

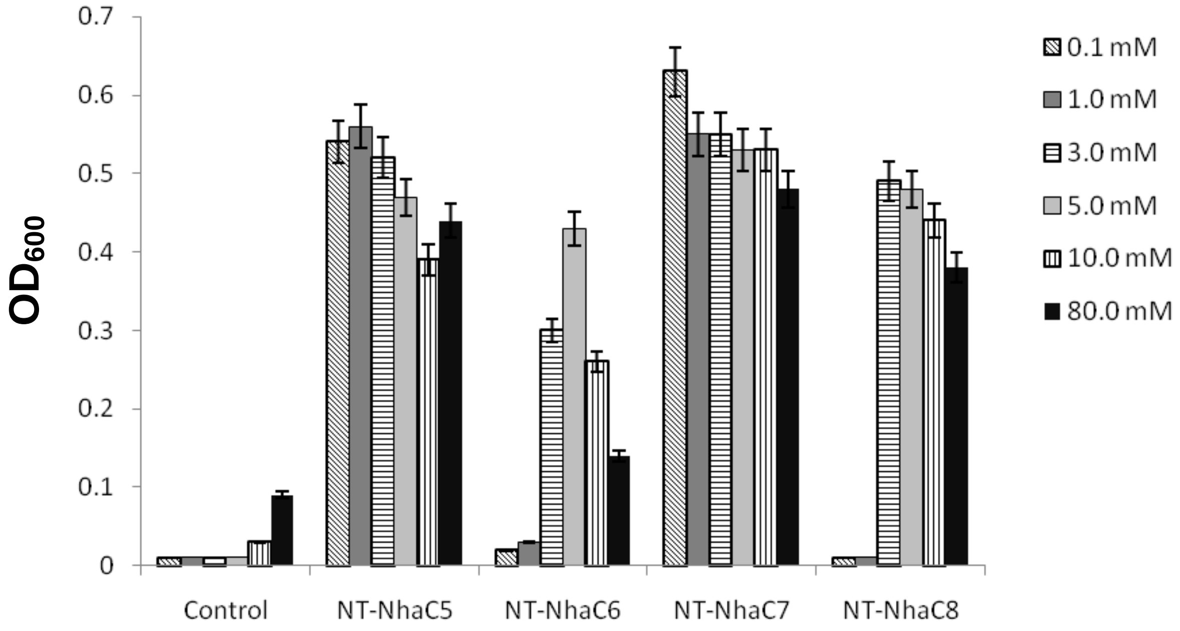

Figure S3. Frequency of isoelectric points in the proteome of *N. thermophilus*. pI values were predicted by the Compute pI/Mw tool on www.expasy.org.

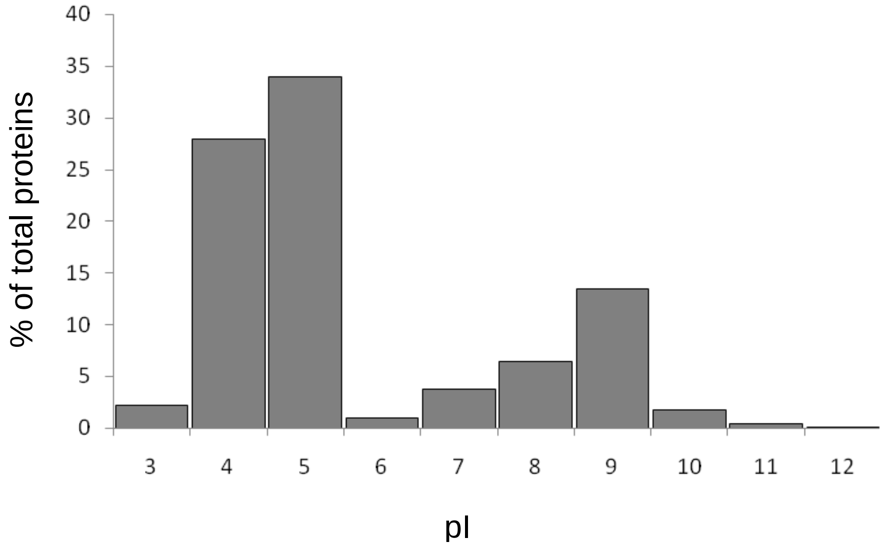

References

Epstein, W. & B. S. Kim, (1971) Potassium transport loci in *Escherichia coli* K-12. *J Bacteriol* **108**: 639-644.

Ito, M., A. Guffanti, J. Zemsky, D. Ivey & T. Krulwich, (1997) Role of the *nhaC*-encoded Na^+/H^+ antiporter of alkaliphilic *Bacillus firmus* OF4. *J Bacteriol* **179**: 3851-3857.

Mesbah, N. M., D. B. Hedrick, A. D. Peacock, M. Rohde & J. Wiegel, (2007) *Natranaerobius thermophilus* gen. nov. sp. nov., a halophilic, alkalithermophilic bacterium from soda lakes of the Wadi An Natrun, Egypt, and proposal of *Natranaerobiaceae* fam. nov. and *Natranaerobiales* ord. nov. *Int J Syst Evol Microbiol* **57**: 2507-2512.

Nozaki, K., K. Inaba, T. Kuroda, M. Tsuda & T. Tsuchiya, (1996) Cloning and sequencing of the gene for the Na^+/H^+ antiporter of *Vibrio parahaemolyticus*. *Biochem Biophys Res Commun* **222**: 774-779.

Olsson, K., S. Keis, H. W. Morgan, P. Dimroth & G. M. Cook, (2003) Bioenergetic properties of the thermoalkaliphilic *Bacillus* sp. strain TA2.A1. *J Bacteriol* **185**: 461-465.

Radchenko, M. V., R. Waditee, s. Oshimi, M. Fukuhara, T. Takabe & T. Nakamura, (2006) Cloning, functional expression and primary characterization of *Vibrio parahaemolyticus* K^+/H^+ antiporter genes in *Escherichia coli*. *Mol Microbiol* **59**: 651-663.

Ren, Q., K. Chen & I. T. Paulsen, (2007) TransportDB: a comprehensive database resource for cytoplasmic membrane transport systems and outer membrane channels. *Nucl Acids Res* **35**: D274-279.

Wei, Y., A. A. Guffanti, M. Ito & T. A. Krulwich, (2000) *Bacillus subtilis* YqkI Is a novel malic/Na^+lactate antiporter that enhances growth on malate at low protonmotive force. *J Biol Chem* **275**: 30287-30292.

Wilson, K., (1997) Preparation of genomic DNA from bacteria. In: Current Protocols in Molecular Biology. F. M. Ausubel, R. Brent, R. E. Kingston, D. D. Moore, J. G. Seidman, J. A. Smith & K. Struhl (eds). John Wiley & Sons, Inc., pp. 2.4.1-2.4.5.
